# Development, Reproducibility, and Validity of a Semiquantitative Food Frequency Questionnaire for Use among the Adult Population in Reunion Island

**DOI:** 10.1016/j.cdnut.2025.107571

**Published:** 2025-10-11

**Authors:** Eric O. Verger, Sabrina Eymard-Duvernay, Sarah Amiri, Noah Nourly, Magali Tarnus, Julie Gauvreau-Béziat, Laure Du Chaffaut, Marine Oseredczuk, Benjamin Allès, Caroline Méjean

**Affiliations:** 1 MoISA, Univ Montpellier, CIRAD, CIHEAM-IAMM, INRAE, IRD, L’Institut Agro, Montpellier, France; 2 Independent Dietician, Lekip Diet, Sainte-Marie, La Réunion, France; 3 ANSES, French Agency for Food, Environmental and Occupational Health & Safety, Risk Assessment Department, Maisons-Alfort, France; 4 Université Sorbonne Paris Nord and Université Paris Cité, Inserm, INRAE, CNAM, Center of Research in Epidemiology and StatisticS (CRESS), Nutritional Epidemiology Research Team (EREN), Bobigny, France

**Keywords:** French overseas region, dietary habits, assessment, tool, food frequency questionnaire

## Abstract

**Background:**

To date, there is no specific food frequency questionnaire (FFQ) to assess dietary habits in any of the French overseas regions.

**Objectives:**

This study aimed to describe the development of a culture and context-specific semiquantitative FFQ for use among the adult population in Reunion Island, to assess its reproducibility and its relative validity compared with 24-h dietary recalls (DRs).

**Methods:**

The CARI (observatoire des Comportements Alimentaires à la RéunIon) FFQ was adapted from the NutriNet-Santé FFQ, with a revised list of 181 food and beverage items reflecting local dietary practices and nutritional concerns. To assess its reproducibility, the FFQ was administered twice, 4 wk apart, to a purposive sample of 108 adults in Reunion Island. During the same period, participants also completed a weekly DR to evaluate the FFQ’s validity. Reproducibility and validity were assessed for 18 food groups, energy, and 38 nutrients using correlations, cross-classification, and weighted κ.

**Results:**

Regarding reproducibility, we found a median rank correlation of 0.56 (nutrients) and 0.64 (food groups). Most participants were correctly classified with a median of 78% (nutrients) and 83% (food group), whereas gross misclassification was low (2.8% and 0.9%, respectively). We found a median weighted κ of 0.44 (nutrients) and 0.47 (food groups). Regarding validity, we found a median crude rank correlation of 0.51 (nutrients) and 0.43 (food groups). Most participants were correctly classified with a median agreement of 71% for nutrients and of 68% for food groups, whereas gross misclassification was low (1.9% and 0.9%, respectively). We found a median weighted κ of 0.32 (nutrients) and 0.27 (food groups).

**Conclusions:**

The CARI FFQ has a moderate to good level of validity for ranking food and nutrient intakes, and a good level of reproducibility, supporting its use for dietary assessment in Reunion Island.

## Introduction

Reunion Island is a small volcanic island with a tropical climate, located in the southern Indian Ocean, between Mauritius and Madagascar. More than 850,000 people live in this 2500 km^2^ territory, nearly half of whom are under the age of 25 [1]. From the 17th to the 21st century, Reunion Island was successively a French colony, a French department, and finally 1 of the 5 French overseas regions [2], which means that French national laws and regulations apply there, with the possibility of adjustments to take into account their specific characteristics. Over the last decades, Reunion Island and the other French overseas regions have experienced more recent and rapid demographic, social, urban, and economic changes than mainland France. These populations have lived through a “compressed modernity,” transitioning in a very short period from an agricultural society to a rapidly evolving consumer society [3]. Related to these changes, French overseas regions have undergone and are still undergoing major changes in their food systems, including the expansion of the supply of processed food [3]. These shifts went along with the development of chronic diseases such as obesity, hypertension, and diabetes, as well as unhealthy dietary patterns with high consumption of sweetened beverages and inadequate intakes of micronutrients [4]. However, data on eating habits and nutritional intakes to characterize the nutritional transition in the French overseas regions remains scarce and incomplete.

To make up for this lack of data [4], the project of an observation center in Reunion Island, named CARI (observatoire des Comportements Alimentaires à la RéunIon; https://pepr-sams.fr/en/2024/03/26/cari-en/), aims to produce monitoring data based on regular large-scale studies on the 4 dimensions of sustainability within food systems: food security and nutrition, environmental, economic, and social [5,6]. The implementation of an observation center on diet and nutrition in Reunion Island requires significant methodological developments to adapt the tools and methods used in the studies conducted in mainland France to the specific population and territorial characteristics of this overseas region. One of the scientific challenges of the CARI project is to develop and validate methods to obtain robust and reliable data on dietary intakes for this territory.

Food frequency questionnaire (FFQ) is a retrospective method providing information on habitual diet by asking about the frequency with which a predetermined list of foods is consumed over a reference period [7,8]. The number of foods on the list varies (from around 20 to >200) and the consumption period usually covers the past 12 mo [9,10]. Although FFQs cannot be relied on to produce accurate estimates of absolute intake of foods, energy, and nutrients compared with less biased short-term instrument [e.g., 24-h dietary recall (DR)], they have been shown to have good validity for ranking food and nutrient intakes within a population, and also distinguish these intakes between subpopulations [9,10]. In addition, due to their ease of administration, relatively low respondent burden, and low cost compared with other dietary assessment methods, FFQs have been extensively used in large-scale studies. As a result, FFQ was determined to be a suitable method to assess dietary intakes in the CARI project.

A substantial body of literature highlights the importance of developing or adapting an FFQ to account for the food culture, dietary habits, and nutritional challenges specific to a given country or population [9,[11], [12], [13], [14]]. A large part of Reunion Island’s food supply is imported, predominantly from mainland France, except for fresh vegetables and fresh meat, which are produced locally [15]. Furthermore, due to its colonial history, several major food cultures coexist in Reunion Island [16]. Although the different FFQ validated for mainland France [12,17] cover part of the diet in Reunion Island, they are not entirely suitable for this context. To the best of our knowledge, no FFQ has been developed specifically for Reunion Island or for other overseas regions. The aims of the current study were: *1*) to describe the development of a culture and context-specific semiquantitative FFQ for use among the adult population in Reunion Island, *2*) to assess its reproducibility, and *3*) to assess its relative validity compared with web-based 24-h DRs.

## Methods

### Development of the CARI FFQ

The layout and format of the CARI FFQ were based on the French FFQ of the NutriNet-Santé study, itself adapted from the SU.VI.MAX FFQ [17]. Unlike the previous FFQs that covered the past 12 mo, the CARI FFQ was designed to measure a participant's usual food intake during the past month. Each food and beverage item of the FFQ represented either a single food (e.g., papaya), a combination of single foods (e.g., zucchinis and eggplants), or a food type further described by examples of single foods (e.g., salted oleaginous fruits such as peanuts, cashews, almonds, pistachios, hazelnuts, walnuts, etc.). We paid particular attention to adapting the food lists of the NutriNet-Santé and SU.VI.MAX FFQs to better reflect the food culture, dietary practices, and nutritional challenges on Reunion Island. Although this involved adding local foods and recipes (e.g., Cari sauce, which is a Creole specialty based on tomatoes and spices, and Chemin de Fer cake, which is a roll cake filled with vanilla cream and covered with red sugar), we also reconsidered whether certain food items should be more or less detailed. For example, we proposed 6 different items for fruit juices instead of just 1 in the NutriNet-Santé FFQ, given that the total sugars, vitamin A, and vitamin C content vary greatly depending on the fruit used. To adapt this list as well as possible, we have worked with local dietitians, used data from the latest household budget survey on Reunion Island [18], data on import, agricultural production and school canteen menus [19] to identify the most purchased and consumed food items and dishes in Reunion Island and how they are prepared, seasoned, and consumed. The food list contained 181 food and beverage items classified into 17 categories. The frequency of intake of each item was assessed within a set of 8 frequency choices for consumption ranging from “never” to “several times per day.” For 107 items, a serving was assigned in terms of units or common portions (e.g., half a papaya) or household measures (e.g., glass, cup, or spoon), and participants can specify the number of servings consumed. For 74 foods and beverages, which are usually not eaten in a predetermined portion size, sets of color photographs from the validated photograph manual developed for the SU.VI.MAX study was used [20]. Participants were asked to provide only 1 answer. Intake of energy and 38 nutrients from the 181 food items was calculated using the NutriNet-Santé food composition table [21]. The food composition table has been extended with 25 foods and recipes from Reunion Island whose nutritional composition has either been borrowed from the French Centre d'Information sur la Qualité des Aliments composition table [22] or calculated according to the EuroFIR recipe calculation procedure [23].

### Validity and reproducibility of the CARI FFQ

When a FFQ is adapted from previously validated FFQs, it is highly recommended to investigate the validity and reproducibility of this new FFQ in a set of individuals representative of the main study target population [7,9,24]. The design and assessment of the validity and reproducibility of the CARI FFQ were based on the recommendations provided by the literature [7,9,24]. The validity of the CARI FFQ was assessed against repeated DRs and its reproducibility using a test–retest methodology.

#### Study sample

To carry out reproducibility and validity analyses, a sample size of ≥50–100 subjects for each demographic group is usually recommended [9,10]. To have a sample size of ≥50 males and 50 females, and allowing for 15% dropout, the recruitment target was 120 adults aged between 18 and 80 who had been living in Reunion Island for ≥ 6 mo, with the aim of obtaining a balanced and diversified sample in terms of gender, age groups, socioprofessional categories (based on the classification of occupations and socioprofessional categories of the National Institute of Statistics and Economic Studies) and geographical areas. From March to May 2024, 128 participants were recruited. Recruitment was carried out through affinity networks of dietitians hired to administer CARI FFQ and DRs. The dietitians were instructed to recruit ∼18 participants with a balanced mix of gender and age groups, to avoid having only similar profiles in terms of education and type of activity, and to avoid recruiting from among their patients. The study was approved by the Comité d’Ethique des Projets de l’INRAE (reference IRB00013805, application CEPR12-2023). Written informed consent was obtained from all participants.

#### Study design

**FIGURE 1:**
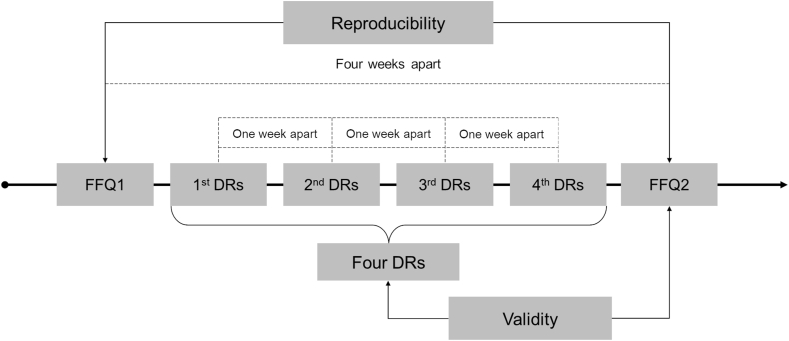
Study design for validity and reproducibility of a semiquantitative food frequency questionnaire for use among the adult population in Reunion Island. Dietitians were instructed: (i) to ideally conduct 1 DR representing a weekend day and 3 DR representing different weekdays, (ii) and to allow a minimum interval of 5 d and a maximum interval of 9 d between 2 DRs. DRs, repeated 24-h dietary recalls; FFQ, food frequency questionnaire.

The study design is presented in Figure 1. Reproducibility of the CARI FFQ was determined by asking participants to complete the questionnaire twice, 4 wk apart. Participants were asked to complete a 24-h DR each week over the same period. Validity was assessed by comparing data evaluated by the second application of the CARI FFQ with data from the 1-mo mean of 4 repeated DRs. Both CARI FFQ and DRs were administered by 7 trained dietitians experienced in interviewing skills. Answers were entered directly onto a tablet with automated quality checks (consistency, completeness) on the NutriNet-Santé study platform (https://etude-nutrinet-sante.fr/).

#### Reference method

Briefly, the DR is a method that relies on a meal-based approach, recording all foods and beverages consumed during a midnight-to-midnight period. First, the participants were asked about all foods and beverages consumed at breakfast, lunch, dinner, and all other eating occasions on the prior day. On the NutriNet-Santé study platform, the DRs use a computer-automated method for entry and coding. Then the participants estimated portion sizes for each reported food and beverage item according to household measures, standard units, or using validated photographs [20]. Intake of energy and 38 nutrients from foods and beverages was calculated using the NutriNet-Santé food composition table [21]. The food composition table has been extended with 25 foods and recipes from Reunion Island whose nutritional composition has either been borrowed from the French Centre d'Information sur la Qualité des Aliments composition table [22] or calculated according to the EuroFIR recipe calculation procedure [23].

### Statistical analysis

Descriptive statistics (median, Q1 and Q3) were calculated for energy intake, 38 nutrients, and 18 food group intakes as estimated by the 2 applications of the FFQ and the average of the DRs. The following indicators of reproducibility were assessed for energy, nutrients, and food groups. Spearman correlation coefficients were calculated to estimate the strength of association of energy, nutrient, and food group intakes as estimated from the 2 applications of the FFQ. Building on previous studies [[25], [26], [27]], we considered correlation coefficients of ≥0.50 as good, 0.40–0.49 as acceptable (higher range of acceptability), 0.20–0.39 as acceptable (lower range of acceptability), and < 0.20 as poor. To assess the capability of the CARI FFQ to correctly rank the intakes at the individual level, absolute intake of energy, nutrients, and food groups was divided into quintiles for the 2 applications of the FFQ. Level of agreement was then determined by a 5 × 5 table, based on exact quintile agreement, exact and adjacent quintile agreement, and on disagreement in extreme quintiles. Building on previous studies [[25], [26], [27]], >70% of subjects correctly classified (exact and adjacent quintiles) and <5% of subjects grossly misclassified (extreme quintiles) were considered as good, between 60% and 70% of subjects correctly classified and <5% of subjects grossly misclassified were considered as acceptable, and >5% of subjects grossly misclassified as poor. We also calculated weighted κ to further quantify the agreement between the 2 applications of the FFQ. We considered values of weighted κ > 0.80 as very good agreement, between 0.61 and 0.80 as good agreement, 0.41 and 0.60 as moderate agreement, 0.21 and 0.40 as fair agreement, and ≤0.20 as poor agreement [28]. We used the same indicators (rank correlation, cross-classification into quintiles, and weighted κ) to assess the validity of the second applications of the FFQ compared with DRs, with some differences as explained afterward. Energy-adjusted Pearson correlation coefficients were calculated to estimate the strength of association of Box-Cox transformed nutrient intakes as estimated from the second application of the FFQ and the average of DRs at the individual level. Energy-adjusted nutrient intakes were estimated using the method of residuals [7]. The observed Pearson correlation coefficients were corrected for attenuation due to within-participant variation in the DR by multiplying them as follows: ${\left\{1+\left(\sigma_{w}^{2}/\sigma_{b}^{2}\right)/m\right\}}^{0.5}$ where *m* represents the number of replicate measurements (*m* = 4) and $\left(\sigma_{w}^{2}/\sigma_{b}^{2}\right)$ is the within-person variance divided by the between-person variance for each nutrient [29]. The within- and between-individual variance components were determined by a mixed effect model with the Box-Cox transformed energy-adjusted intake as the dependent variable and subject as a random intercept. Spearman correlation coefficients were calculated to estimate the strength of association of alcohol and food group intakes as estimated from the second application of the FFQ and the average of the DRs at the individual level (due to non-normal distributions resulting from partial nonconsumption within the sample). We performed sensitivity analysis excluding participants who reported extreme energy intakes (<800 kcal/d or >4200 kcal/d for males and <600 kcal/d or >3500 kcal/d for females) from the FFQ or the average of DRs, as suggested by Subar et al. [24]. All statistical analyses were performed using R Statistical Software (v4.4.0; R Core Team 2024).

## Results

### Description of the participants

Among the 128 participants who were included in the study, 108 completed both the FFQ and the 4 DRs. None of the FFQs had missing data on >10% of FFQ food items. Among the 108 participants, the median age was 40 y (IQR: 29–56) , 51% of participants were female, with a diversity of socioprofessional categories and a good geographical coverage of Reunion Island (Table 1). For sensitivity analysis, we excluded 21 participants who reported extreme energy intakes in the 2 applications of the FFQ, or the second FFQ and the 4 DRs, which led to an analytical sample of 87 participants. There was no difference in the characteristics of the participants excluded.

**TABLE 1:** Demographic characteristics of the study participants: a convenience sample of adults living in Reunion Island (*n* = 108)

Characteristic	Female *N* = 55	Male *N* = 53	Overall *N* = 108
Age, median (Q1, Q3)^1^	41 (28, 58)	37 (29, 54)	40 (29, 56)
Socioprofessional categories, *n* (%)			
Farmers	0	1 (2%)	1 (1%)
Craftsmen, shopkeepers and company directors	6 (11%)	5 (9%)	11 (10%)
Managerial staff	8 (15%)	10 (19%)	18 (17%)
Intermediate professions	9 (16%)	6 (11%)	15 (14%)
Employees	9 (16%)	8 (15%)	17 (16%)
Manual workers	0	5 (9%)	5 (5%)
Retired	10 (18%)	7 (13%)	17 (16%)
Other people not in employment	13 (24%)	11 (21%)	24 (22%)
Region, *n* (%)			
North	22 (40%)	22 (42%)	44 (41%)
West	7 (13%)	8 (15%)	15 (14%)
South	17 (31%)	13 (25%)	30 (28%)
South-West	9 (16%)	10 (19%)	19 (18%)

1 The minimum and maximum ages of the females were 18 and 81, respectively, and for males, 19 and 75.

### Reproducibility

Measures of reproducibility of the CARI FFQ are presented in Table 2 for nutrients and Table 3 for food groups. Median intakes of energy, nutrients, and food groups reported during the first application of the CARI FFQ questionnaire were higher than those reported during the second application, except for 3 food groups (alcoholic beverages, soft beverages, and fruit juices).

**TABLE 2:** Measures of reproducibility of the first application of the CARI food frequency questionnaire (FFQ 1) compared with the second application (FFQ 2) in terms of daily nutrient intakes among adults living in Reunion Island (*n* = 108)

	FFQ 1 median (Q1–Q3)	FFQ 2 median (Q1–Q3)	Spearman correlation coeff. (95% CI)	Intraclass correlation coeff. (95% CI)	Cross-classification^1^			
					% Exact quintiles	% Exact and adjacent quintiles	% Opposite quintiles	Weighted κ (95% CI)
Energy intake (kcal/d)	2291.0 (1665.0–2893.4)	2008.9 (1403.4–2626.4)	0.66 (0.53, 0.78)	0.59 (0.45, 0.70)	39.8	80.6	0.9	0.46 (0.33, 0.56)
Protein (g/d)	87.8 (59.3–123.6)	71.2 (46.9–107.0)	0.65 (0.49, 0.77)	0.62 (0.49, 0.72)	38.9	84.3	3.7	0.47 (0.35, 0.57)
Total carbohydrates (g/d)	221.8 (160.5–282.1)	183.8 (132.2–241.0)	0.66 (0.49, 0.78)	0.58 (0.44, 0.69)	47.2	83.3	1.9	0.51 (0.39, 0.62)
Fiber (g/d)	22.3 (15.9–30.6)	17.3 (12.4–24.9)	0.51 (0.33, 0.67)	0.50 (0.35, 0.63)	37.0	73.2	3.7	0.35 (0.22, 0.47)
Starches (g/d)	124.8 (84.7–168.3)	100.5 (74.9–138.0)	0.60 (0.45, 0.73)	0.55 (0.40, 0.67)	45.4	75.9	0.0	0.46 (0.33, 0.57)
Total sugars (g/d)	84.3 (58.5–114.8)	67.6 (49.4–93.6)	0.68 (0.53, 0.79)	0.61 (0.47, 0.71)	43.5	81.5	1.9	0.48 (0.35, 0.58)
Added sugars (g/d)	32.2 (17.3–55.9)	28.0 (16.0–47.4)	0.75 (0.64, 0.83)	0.73 (0.63, 0.81)	47.2	86.1	0.0	0.56 (0.45, 0.65)
Total fat (g/d)	104.3 (79.5–145.8)	99.7 (69.3–132.8)	0.58 (0.43, 0.70)	0.53 (0.38, 0.65)	33.3	76.9	0.9	0.40 (0.28, 0.50)
PUFAs (g/d)	20.2 (14.5–29.2)	19.3 (11.8–28.4)	0.57 (0.42, 0.71)	0.55 (0.40, 0.67)	42.6	78.7	0.9	0.44 (0.31, 0.56)
MUFAs (g/d)	45.8 (30.9–61.7)	41.6 (26.1–55.1)	0.56 (0.41, 0.69)	0.49 (0.33, 0.62)	36.1	73.2	1.9	0.37 (0.25, 0.49)
SFAs (g/d)	34.5 (21.5–45.8)	27.8 (18.6–37.1)	0.67 (0.53, 0.78)	0.63 (0.50, 0.73)	41.7	85.2	0.9	0.51 (0.39, 0.61)
*n*−3 fatty acids (g/d)	1.5 (1.1–2.1)	1.3 (0.9–1.8)	0.40 (0.20, 0.56)	0.31 (0.13, 0.47)	35.2	68.5	4.6	0.28 (0.15, 0.42)
*n*−6 fatty acids (g/d)	17.3 (12.2–26.5)	16.4 (10.1–24.6)	0.59 (0.43, 0.71)	0.56 (0.42, 0.68)	46.3	76.9	1.9	0.46 (0.32, 0.57)
Cholesterol (mg/d)	329.6 (233.4–495.2)	281.3 (158.5–422.1)	0.69 (0.55, 0.79)	0.69 (0.57, 0.77)	42.6	86.1	1.9	0.51 (0.40, 0.61)
Alcohol (g/d)	4.6 (0.1–13.1)	3.8 (0.6–11.6)	0.80 (0.68, 0.89)	0.82 (0.75, 0.87)	59.3	91.7	0.0	0.60 (0.48, 0.71)
Retinol (μg/d)	305.0 (197.0–476.8)	240.6 (157.7–385.0)	0.62 (0.46, 0.75)	0.62 (0.48, 0.72)	36.1	81.5	2.8	0.43 (0.32, 0.54)
β-carotene (μg/d)	4719.8 (2010.8–7543.9)	3520.6 (2158.8–6149.6)	0.66 (0.51, 0.77)	0.64 (0.52, 0.74)	41.7	81.5	1.9	0.48 (0.36, 0.58)
Thiamine (mg/d)	1.3 (0.9–1.6)	1.0 (0.7–1.4)	0.60 (0.43, 0.73)	0.52 (0.36, 0.64)	37.0	76.9	2.8	0.41 (0.28, 0.52)
Riboflavin (mg/d)	1.5 (1.1–1.9)	1.2 (0.9–1.8)	0.66 (0.52, 0.79)	0.59 (0.46, 0.70)	38.9	85.2	1.9	0.48 (0.36, 0.58)
Niacin (mg/d)	21.1 (15.5–29.0)	17.8 (12.8–27.4)	0.58 (0.41, 0.73)	0.55 (0.41, 0.67)	41.7	82.4	2.8	0.46 (0.33, 0.57)
Vitamin B5 (mg/d)	5.4 (4.0–7.0)	4.3 (3.1–6.5)	0.63 (0.46, 0.75)	0.58 (0.45, 0.70)	36.1	86.1	3.7	0.46 (0.34, 0.56)
Vitamin B6 (mg/d)	1.9 (1.3–2.6)	1.5 (1.1–2.4)	0.61 (0.44, 0.75)	0.56 (0.42, 0.68)	40.7	82.4	3.7	0.46 (0.33, 0.57)
Folates (μg/d)	319.4 (231.6–441.4)	259.6 (190.1–349.4)	0.53 (0.34, 0.68)	0.52 (0.36, 0.64)	41.7	77.8	3.7	0.41 (0.28, 0.53)
Vitamin B12 (μg/d)	4.5 (2.8–6.0)	3.5 (2.3–5.5)	0.60 (0.43, 0.73)	0.64 (0.51, 0.74)	44.4	76.9	1.9	0.46 (0.33, 0.57)
Vitamin C (mg/d)	112.1 (67.3–147.5)	90.4 (59.8–133.7)	0.56 (0.38, 0.72)	0.55 (0.40, 0.67)	41.7	81.5	3.7	0.44 (0.31, 0.55)
Vitamin D (μg/d)	2.7 (1.9–3.9)	2.2 (1.4–3.4)	0.60 (0.44, 0.73)	0.61 (0.47, 0.71)	44.4	76.9	2.8	0.46 (0.33, 0.57)
Vitamin E (mg/d)	19.3 (14.3–29.5)	18.2 (11.5–28.2)	0.55 (0.38, 0.69)	0.52 (0.37, 0.65)	39.8	75.0	0.9	0.40 (0.27, 0.51)
Vitamin K1 (μg/d)	167.1 (92.9–318.6)	157.0 (81.9–241.8)	0.55 (0.38, 0.68)	0.57 (0.43, 0.69)	40.7	75.9	2.8	0.40 (0.27, 0.52)
Calcium (mg/d)	789.5 (607.9–1053.1)	648.8 (458.3–845.4)	0.60 (0.44, 0.72)	0.52 (0.37, 0.64)	39.8	82.4	0.9	0.46 (0.34, 0.56)
Magnesium (mg/d)	383.3 (305.6–527.9)	314.9 (227.5–436.0)	0.60 (0.46, 0.73)	0.49 (0.34, 0.62)	41.7	77.8	3.7	0.42 (0.29, 0.53)
Phosphorus (mg/d)	1361.0 (1018.2–1854.1)	1094.8 (778.8–1625.5)	0.61 (0.44, 0.75)	0.55 (0.41, 0.67)	35.2	80.6	2.8	0.42 (0.30, 0.54)
Potassium (mg/d)	2970.4 (2238.8–4107.5)	2452.2 (1789.7–3345.8)	0.58 (0.41, 0.72)	0.51 (0.36, 0.64)	36.1	82.4	3.7	0.43 (0.31, 0.54)
Sodium (mg/d)	2302.5 (1511.9–3058.2)	1788.2 (1287.3–2672.2)	0.68 (0.53, 0.80)	0.59 (0.45, 0.70)	47.2	87.0	2.8	0.55 (0.44, 0.65)
Copper (mg/d)	1.7 (1.3–2.3)	1.4 (1.0–1.9)	0.56 (0.38, 0.69)	0.49 (0.34, 0.62)	40.7	76.9	1.9	0.42 (0.29, 0.53)
Iron (mg/d)	14.5 (11.3–19.6)	12.2 (8.8–17.2)	0.54 (0.37, 0.68)	0.47 (0.31, 0.61)	38.0	78.7	2.8	0.40 (0.27, 0.51)
Iodine (μg/d)	99.3 (76.5–134.2)	84.6 (61.0–111.8)	0.61 (0.44, 0.73)	0.56 (0.41, 0.68)	42.6	79.6	2.8	0.47 (0.35, 0.58)
Manganese (mg/d)	4.4 (3.2–6.5)	3.3 (2.5–5.1)	0.57 (0.41, 0.71)	0.51 (0.36, 0.64)	39.8	79.6	2.8	0.43 (0.30, 0.54)
Selenium (μg/d)	79.6 (59.0–110.8)	68.9 (49.6–96.2)	0.59 (0.42, 0.73)	0.55 (0.40, 0.67)	38.9	78.7	3.7	0.41 (0.28, 0.53)
Zinc (mg/d)	10.7 (7.9–14.9)	8.8 (6.6–13.2)	0.58 (0.40, 0.72)	0.56 (0.41, 0.67)	38.0	77.8	2.8	0.41 (0.28, 0.52)

Abbreviation: CI, confidence interval.

1 Level of agreement was then determined by a 5 × 5 table of FFQs quintile, based on exact quintile agreement, exact and adjacent quintile agreement, and on disagreement in extreme quintiles.

**TABLE 3:** Measures of reproducibility of the first application of the CARI food frequency questionnaire (FFQ 1) compared with the second application (FFQ 2) in terms of daily food groups intakes (in grams) among adults living in Reunion Island (*n* = 108)

	FFQ 1 median (Q1–Q3)	FFQ 2 median (Q1–Q3)	Spearman correlation coeff. (95% CI)	Cross-classification^1^			
				% Exact quintiles	% Exact and adjacent quintiles	% Opposite quintiles	Weighted κ (95% CI)
Alcoholic beverages	62.1 (1.3–181.0)	64.4 (9.0–132.9)	0.83 (0.72, 0.90)	62.0	92.6	0.0	0.63 (0.52, 0.73)
Unsweetened beverages	851.1 (614.0–1319.4)	818.2 (582.7–1119.3)	0.41 (0.23, 0.56)	32.4	69.4	2.8	0.29 (0.16, 0.42)
Soft drinks and fruit juices	47.1 (10.8–143.2)	47.1 (10.8–117.9)	0.70 (0.56, 0.81)	41.7	88.0	0.9	0.49 (0.36, 0.58)
Cereal products	226.5 (113.8–318.9)	181.1 (100.5–270.8)	0.69 (0.54, 0.80)	45.4	82.4	0.9	0.50 (0.38, 0.62)
Wholegrain cereal products	15.7 (1.8–48.4)	9.0 (0.0–40.4)	0.55 (0.39, 0.69)	50.9	83.3	0.0	0.45 (0.31, 0.57)
Fruits	168.8 (74.2–272.3)	130.8 (51.5–261.5)	0.65 (0.48, 0.78)	47.2	83.3	1.9	0.51 (0.39, 0.62)
Milk and dairy products	44.0 (23.9–89.5)	30.5 (14.8–71.9)	0.72 (0.57, 0.82)	55.6	85.2	0.0	0.59 (0.48, 0.70)
Vegetables	195.2 (102.9–384.4)	165.2 (114.0–286.4)	0.51 (0.35, 0.66)	36.1	75.9	3.7	0.36 (0.23, 0.48)
Pulses	23.5 (6.0–55.0)	17.9 (6.9–40.2)	0.60 (0.45, 0.72)	43.5	80.6	0.0	0.47 (0.35, 0.58)
Fats	38.3 (24.8–54.2)	36.2 (22.3–51.9)	0.45 (0.27, 0.58)	26.9	72.2	1.9	0.28 (0.16, 0.40)
Nuts and seeds	5.7 (1.3–12.3)	3.3 (0.0–11.0)	0.68 (0.54, 0.79)	53.7	88.0	0.0	0.52 (0.39, 0.63)
Eggs	25.0 (13.7–42.9)	21.0 (11.9–42.0)	0.61 (0.46, 0.73)	32.4	79.6	1.9	0.40 (0.27, 0.50)
Fish and fishery products	33.0 (18.5–47.2)	26.1 (16.2–48.7)	0.63 (0.47, 0.77)	40.7	79.6	2.8	0.46 (0.33, 0.57)
Sweet or sweet and fat products	100.4 (61.8–166.5)	85.1 (52.2–123.1)	0.68 (0.55, 0.78)	44.4	83.3	0.0	0.51 (0.40, 0.62)
Sauces	15.1 (8.1–30.5)	14.0 (5.9–26.4)	0.62 (0.48, 0.74)	42.6	75.0	0.9	0.46 (0.34, 0.56)
Tubers	35.6 (19.1–68.9)	27.9 (12.1–51.4)	0.54 (0.37, 0.68)	41.7	75.9	0.9	0.42 (0.29, 0.54)
Meats	98.8 (50.1–168.4)	78.3 (39.8–152.3)	0.70 (0.55, 0.82)	46.3	87.0	0.9	0.55 (0.44, 0.65)
Processed meats	33.1 (16.7–59.8)	25.0 (11.5–45.1)	0.68 (0.54, 0.79)	42.6	84.3	2.8	0.49 (0.37, 0.60)

Abbreviation: CI, confidence interval.

1 Level of agreement was then determined by a 5 × 5 table of FFQs quintile, based on exact quintile agreement, exact and adjacent quintile agreement, and on disagreement in extreme quintiles.

We found a median rank correlation of 0.56 (from 0.31 to 0.82) for nutrients (Table 2) and 0.64 (from 0.41 to 0.83) for food groups (Table 3). The correlation coefficients were considered as good for 34 nutrients, and acceptable for 5 nutrients (with 4 in the higher range of acceptability and 1 in the lower range). The correlation coefficients were considered as good for all food groups, except 2, which were considered in the higher range of acceptability (unsweetened beverages and fats).

Most participants were correctly classified into the same or adjacent quintile (median of 78% for nutrients and 83% for food groups), whereas the median percentage of gross misclassification was low (2.8% for nutrients and 0.9% for food groups). The reproducibility of the CARI FFQ in ranking the nutrient intakes at the individual level was considered good for all the nutrients, except 1, which was considered as acceptable (*n*−3 fatty acids). Similarly, it was considered as good for all food groups, except 1, which was considered acceptable (unsweetened beverages).

We found a median weighted κ of 0.44 (from 0.28 to 0.60) for nutrients (Table 2) and 0.47 (from 0.29 to 0.63) for food groups (Table 3). The level of agreement from weighted κ was considered as moderate for 32 nutrients and fair for 7 nutrients. The level of agreement from weighted κ was considered as good for 1 food group, moderate for 14 food groups, and fair for 3 food groups.

Sensitivity analyses excluding participants who reported extreme energy intakes produced similar results (Supplemental Tables 1 and 2).

### Validity

Measures of validity of the CARI FFQ compared with DRs are presented in Table 4 for nutrients and Table 5 for food groups.

**TABLE 4:** Measures of validity of the second application of the CARI food frequency questionnaire (FFQ 2) compared with the average of 24-h dietary recalls (DRs) in terms of daily nutrient intakes among adults living in Reunion Island (*n* = 108)

	FFQ 2 median (Q1–Q3)	FFQ 2 Box-Cox transformation λ parameter	DRs median (Q1–Q3)	DRs Box-Cox transformation λ parameter	Spearman correlation coeff. (95% CI)	Deattenuated^1^ energy-adjusted Pearson correlation coeff. (95% CI)	Cross-classification^2^			
							% Exact quintiles	% Exact and adjacent quintiles	% Opposite quintiles	Weighted κ (95% CI)
Energy intake (kcal/d)	2008.9 (1403.4–2626.4)	0.130	1604.3 (1241.1–2008.5)	–0.016	0.60 (0.45, 0.71)	0.66 (0.53, 0.75)	38.9	75.9	0.9	0.41 (0.28, 0.52)
Protein (g/d)	71.2 (46.9–107.0)	0.151	63.6 (50.2–79.5)	0.196	0.60 (0.45, 0.71)	0.41 (0.25, 0.56)	36.1	73.2	0.9	0.37 (0.25, 0.49)
Total carbohydrates (g/d)	183.8 (132.2–241.0)	0.018	167.0 (126.2–204.3)	–0.044	0.61 (0.47, 0.72)	0.42 (0.26, 0.57)	38.9	78.7	1.9	0.43 (0.31, 0.54)
Fiber (g/d)	17.3 (12.4–24.9)	0.113	13.0 (10.8–18.0)	0.192	0.46 (0.30, 0.59)	0.66 (0.54, 0.75)	34.3	68.5	3.7	0.32 (0.19, 0.44)
Starches (g/d)	100.5 (74.9–138.0)	0.090	93.0 (73.0–127.1)	0.308	0.57 (0.41, 0.70)	0.37 (0.20, 0.53)	38.9	74.1	1.9	0.40 (0.27, 0.52)
Total sugars (g/d)	67.6 (49.4–93.6)	0.071	63.8 (46.6–86.6)	0.241	0.54 (0.39, 0.67)	0.53 (0.38, 0.66)	41.7	75.0	0.0	0.42 (0.29, 0.54)
Added sugars (g/d)	28.0 (16.0–47.4)	0.161	33.9 (20.6–50.3)	0.378	0.68 (0.57, 0.77)	0.59 (0.45, 0.70)	35.2	78.7	0.0	0.44 (0.32, 0.55)
Total fat (g/d)	99.7 (69.3–132.8)	0.128	70.1 (52.8–87.4)	0.232	0.42 (0.25, 0.57)	0.24 (0.05, 0.41)	28.7	65.7	1.9	0.27 (0.15, 0.39)
PUFAs (g/d)	19.3 (11.8–28.4)	–0.063	10.7 (8.9–15.2)	0.124	0.27 (0.06, 0.46)	0.18 (0.00, 0.36)	29.6	64.8	4.6	0.21 (0.07, 0.34)
MUFAs (g/d)	41.6 (26.1–55.1)	0.206	27.5 (21.2–33.4)	0.299	0.39 (0.22, 0.56)	0.25 (0.06, 0.42)	22.2	63.9	2.8	0.20 (0.08, 0.32)
SFAs (g/d)	27.8 (18.6–37.1)	0.068	25.6 (19.3–34.6)	0.266	0.54 (0.38, 0.66)	0.49 (0.33, 0.62)	35.2	72.2	0.9	0.37 (0.24, 0.49)
*n*−3 fatty acids (g/d)	1.3 (0.9–1.8)	0.032	0.9 (0.7–1.2)	–0.033	0.28 (0.08, 0.47)	0.25 (0.07, 0.42)	29.6	62.0	4.6	0.19 (0.05, 0.33)
*n*−6 fatty acids (g/d)	16.4 (10.1–24.6)	–0.090	9.1 (7.2–13.4)	0.130	0.27 (0.05, 0.44)	0.22 (0.04, 0.40)	31.5	61.1	5.6	0.19 (0.05, 0.33)
Cholesterol (mg/d)	281.3 (158.5–422.1)	0.248	284.9 (217.1–347.5)	0.220	0.61 (0.46, 0.72)	0.47 (0.31, 0.60)	40.7	74.1	0.0	0.42 (0.29, 0.54)
Alcohol (g/d)	3.8 (0.6–11.6)	—	2.4 (0.0–10.2)	—	0.66 (0.50, 0.78)	—	35.2	92.6	0.0	0.38 (0.27, 0.48)
Retinol (μg/d)	240.6 (157.7–385.0)	0.035	244.7 (176.3–360.3)	0.190	0.49 (0.32, 0.63)	0.41 (0.23, 0.55)	32.4	72.2	1.9	0.31 (0.18, 0.43)
β-carotene (μg/d)	3520.6 (2158.8–6149.6)	0.121	1863.7 (1225.9–3318.5)	0.082	0.45 (0.28, 0.60)	0.49 (0.33, 0.62)	26.9	69.4	1.9	0.27 (0.14, 0.40)
Thiamine (mg/d)	1.0 (0.7–1.4)	0.055	0.8 (0.6–0.9)	0.010	0.40 (0.21, 0.55)	0.47 (0.31, 0.61)	31.5	71.3	5.6	0.28 (0.15, 0.41)
Riboflavin (mg/d)	1.2 (0.9–1.8)	0.071	1.2 (0.8–1.4)	0.232	0.51 (0.34, 0.64)	0.45 (0.29, 0.59)	37.0	69.4	2.8	0.34 (0.20, 0.46)
Niacin (mg/d)	17.8 (12.8–27.4)	–0.003	14.5 (11.1–19.0)	0.177	0.54 (0.36, 0.67)	0.49 (0.33, 0.62)	32.4	67.6	0.0	0.33 (0.20, 0.45)
Vitamin B5 (mg/d)	4.3 (3.1–6.5)	0.086	4.0 (3.1–4.9)	0.164	0.56 (0.41, 0.69)	0.52 (0.37, 0.65)	38.9	72.2	0.9	0.39 (0.25, 0.51)
Vitamin B6 (mg/d)	1.5 (1.1–2.4)	0.070	1.3 (0.9–1.5)	0.203	0.55 (0.40, 0.68)	0.58 (0.43, 0.69)	35.2	72.2	0.0	0.37 (0.25, 0.49)
Folates (μg/d)	259.6 (190.1–349.4)	0.049	207.2 (162.5–259.2)	0.235	0.43 (0.26, 0.59)	0.50 (0.34, 0.63)	33.3	66.7	2.8	0.31 (0.17, 0.43)
Vitamin B12 (μg/d)	3.5 (2.3–5.5)	0.227	2.9 (1.9–4.0)	0.062	0.47 (0.29, 0.61)	0.35 (0.18, 0.51)	32.4	71.3	0.9	0.32 (0.18, 0.44)
Vitamin C (mg/d)	90.4 (59.8–133.7)	–0.149	50.1 (39.1–74.5)	0.321	0.47 (0.29, 0.61)	0.52 (0.36, 0.64)	30.6	67.6	1.9	0.28 (0.15, 0.41)
Vitamin D (μg/d)	2.2 (1.4–3.4)	0.141	1.8 (1.3–2.7)	0.196	0.51 (0.34, 0.64)	0.40 (0.23, 0.55)	30.6	69.4	0.9	0.32 (0.19, 0.44)
Vitamin E (mg/d)	18.2 (11.5–28.2)	–0.147	9.8 (7.7–12.8)	0.131	0.28 (0.07, 0.46)	0.27 (0.08, 0.44)	27.8	64.8	4.6	0.20 (0.06, 0.33)
Vitamin K1 (μg/d)	157.0 (81.9–241.8)	0.075	85.2 (43.6–139.1)	–0.111	0.44 (0.26, 0.59)	0.47 (0.31, 0.61)	34.3	71.3	1.9	0.33 (0.19, 0.45)
Calcium (mg/d)	648.8 (458.3–845.4)	–0.106	640.2 (492.4–856.7)	0.168	0.52 (0.35, 0.66)	0.38 (0.20, 0.53)	32.4	75.9	1.9	0.36 (0.23, 0.48)
Magnesium (mg/d)	314.9 (227.5–436.0)	0.143	255.8 (194.7–324.7)	–0.030	0.56 (0.39, 0.70)	0.62 (0.49, 0.73)	38.9	74.1	1.9	0.40 (0.27, 0.52)
Phosphorus (mg/d)	1094.8 (778.8–1625.5)	0.160	964.7 (786.5–1282.6)	0.150	0.57 (0.44, 0.69)	0.37 (0.19, 0.52)	37.0	74.1	1.9	0.40 (0.27, 0.51)
Potassium (mg/d)	2452.2 (1789.7–3345.8)	0.042	2146.9 (1724.0–2571.7)	0.257	0.51 (0.33, 0.65)	0.61 (0.47, 0.71)	34.3	69.4	0.9	0.32 (0.18, 0.44)
Sodium (mg/d)	1788.2 (1287.3–2672.2)	–0.024	2464.0 (1833.3–3062.3)	0.317	0.47 (0.31, 0.62)	0.13 (–0.06, 0.31)	32.4	72.2	3.7	0.32 (0.18, 0.44)
Copper (mg/d)	1.4 (1.0–1.9)	0.089	1.3 (1.0–1.6)	–0.145	0.49 (0.32, 0.63)	0.57 (0.43, 0.69)	32.4	68.5	1.9	0.31 (0.18, 0.43)
Iron (mg/d)	12.2 (8.8–17.2)	0.211	10.7 (8.0–13.4)	–0.031	0.41 (0.23, 0.58)	0.45 (0.29, 0.59)	31.5	66.7	3.7	0.26 (0.12, 0.39)
Iodine (μg/d)	84.6 (61.0–111.8)	0.053	110.4 (76.8–143.9)	–0.267	0.42 (0.23, 0.57)	0.26 (0.07, 0.43)	30.6	68.5	4.6	0.28 (0.15, 0.41)
Manganese (mg/d)	3.3 (2.5–5.1)	–0.074	3.0 (2.1–4.0)	0.007	0.54 (0.39, 0.67)	0.67 (0.54, 0.76)	36.1	75.9	1.9	0.39 (0.26, 0.50)
Selenium (μg/d)	68.9 (49.6–96.2)	0.047	71.5 (60.4–91.8)	0.292	0.35 (0.16, 0.50)	0.37 (0.20, 0.52)	25.0	64.8	2.8	0.19 (0.05, 0.32)
Zinc (mg/d)	8.8 (6.6–13.2)	0.090	8.0 (6.1–9.6)	0.179	0.51 (0.35, 0.64)	0.31 (0.13, 0.47)	36.1	70.4	0.9	0.35 (0.22, 0.46)

Abbreviation: CI, confidence interval.

1 Pearson correlation coefficients were corrected for attenuation based on the number of replicate measurements, the within-person variance and the between-person variance for each nutrient (see Method section for more details).

2 Level of agreement was then determined by a 5 × 5 table of FFQ quintile by average of the DRs quintile, based on exact quintile agreement, exact and adjacent quintile agreement, and on disagreement in extreme quintiles.

**TABLE 5:** Measures of validity of the second application of the CARI food frequency questionnaire (FFQ 2) compared with 24-h dietary recalls (DRs) in terms of daily food groups intakes (in grams) among adults living in Reunion Island (*n* = 108)

	FFQ 2 median (Q1–Q3)	DRs median (Q1–Q3)	Spearman correlation coeff. (95% CI)	Cross-classification^1^			
				% Exact quintiles	% Exact and adjacent quintiles	% Opposite quintiles	Weighted κ (95% CI)
Alcoholic beverages	64.4 (9.0–132.9)	37.5 (0.0–178.8)	0.71 (0.58, 0.80)	31.5	93.5	0.0	0.37 (0.26, 0.46)
Unsweetened beverages	818.2 (582.7, 1119.3)	1616.2 (1224.2, 2021.2)	0.09 (–0.09, 0.27)	18.5	52.8	5.6	0.02 (–0.10, 0.14)
Soft drinks and fruit juices	47.1 (10.8–117.9)	43.8 (0.0–174.1)	0.58 (0.44, 0.71)	29.6	68.5	0.0	0.24 (0.14, 0.33)
Cereal products	181.1 (100.5–270.8)	179.9 (128.7–250.2)	0.60 (0.47, 0.71)	33.3	76.9	0.0	0.40 (0.28, 0.51)
Wholegrain cereal products	9.0 (0.0–40.4)	0.0 (0.0–23.2)	0.49 (0.32, 0.65)	30.6	80.6	0.0	0.26 (0.15, 0.36)
Fruits	130.8 (51.5–261.5)	55.5 (8.6–123.4)	0.66 (0.52, 0.77)	33.3	85.2	0.0	0.43 (0.32, 0.52)
Milk and dairy products	30.5 (14.8–71.9)	71.1 (33.4–138.4)	0.40 (0.23, 0.54)	25.9	62.0	2.8	0.20 (0.07, 0.33)
Vegetables	165.2 (114.0–286.4)	124.0 (82.6–178.1)	0.43 (0.26, 0.58)	33.3	68.5	3.7	0.29 (0.16, 0.42)
Pulses	17.9 (6.9–40.2)	19.4 (0.0–41.1)	0.42 (0.25, 0.58)	33.3	75.0	0.0	0.30 (0.18, 0.41)
Fats	36.2 (22.3–51.9)	14.1 (9.5–20.6)	–0.03 (–0.23, 0.17)	18.5	53.7	10.2	–0.01 (–0.14, 0.12)
Nuts and seeds	3.3 (0.0–11.0)	0.0 (0.0–3.5)	0.48 (0.32, 0.63)	31.5	88.0	0.0	0.29 (0.17, 0.39)
Eggs	21.0 (11.9–42.0)	15.7 (3.4–29.8)	0.40 (0.23, 0.57)	31.5	65.7	0.9	0.24 (0.10, 0.37)
Fish and fishery products	26.1 (16.2–48.7)	25.8 (4.6–43.2)	0.36 (0.18, 0.53)	27.8	64.8	4.6	0.21 (0.08, 0.34)
Sweet or sweet and fat products	85.1 (52.2–123.1)	90.4 (56.9–154.0)	0.46 (0.30, 0.60)	31.5	66.7	1.9	0.31 (0.17, 0.42)
Sauces	14.0 (5.9–26.4)	16.1 (8.0–26.0)	0.20 (0.01, 0.38)	27.8	67.6	6.5	0.18 (0.04, 0.31)
Tubers	27.9 (12.1–51.4)	15.6 (0.0–49.9)	0.44 (0.26, 0.59)	35.2	70.4	0.9	0.31 (0.18, 0.42)
Meats	78.3 (39.8–152.3)	75.6 (42.2–114.9)	0.55 (0.40, 0.69)	32.4	72.2	0.9	0.35 (0.22, 0.47)
Processed meats	25.0 (11.5–45.1)	16.3 (5.0–31.4)	0.38 (0.21, 0.54)	27.8	64.8	3.7	0.24 (0.11, 0.36)

Abbreviation: CI, confidence interval.

1 Level of agreement was then determined by a 5 × 5 table of FFQ quintile by average of the DRs quintile, based on exact quintile agreement, exact and adjacent quintile agreement, and on disagreement in extreme quintiles.

Median intakes of energy, nutrients and food groups reported during the second application of the CARI FFQ questionnaire were higher than those reported during the DRs, except for 7 nutrients (added sugars, cholesterol, retinol, vitamin B2, sodium, iodine and selenium) and 5 food groups (unsweetened beverages, milk and dairy products, pulses, sweet or sweet and fat products, and sauces).

We found a median crude rank correlation of 0.51 (from 0.27 to 0.68) for nutrients (Table 4) and 0.43 (from –0.02 to 0.71) for food groups (Table 5). The crude correlation coefficients were considered good for 20 nutrients and acceptable for 19 nutrients, with 13 in the higher range of acceptability and 6 in the lower range. The crude correlation coefficients were considered as good for 5 food groups, acceptable for 11 food groups (with 8 in the higher range of acceptability and 3 in the lower range), and poor for 2 food groups (unsweetened beverages and fats). Energy adjustment and correction for attenuation decreased the correlation coefficients, resulting in a median rank correlation of 0.41 (from 0.13 to 0.67) for nutrients. The energy-adjusted and deattenuated coefficients were considered as good for 12 nutrients, acceptable for 23 nutrients (with 12 in the higher range of acceptability and 11 in the lower range), and poor for 2 nutrients (polyunsaturated fatty acids and sodium).

Most participants were correctly classified into the same or adjacent quintile (median of 71% for nutrients and 68% for food groups), whereas the median percentage of gross misclassification was low (1.9% for nutrients and 0.9% for food groups). The capability of the CARI FFQ to correctly rank the nutrient intakes at the individual level was considered as good for 19 nutrients, acceptable for 17 nutrients and poor for 2 nutrients (*n*−6 fatty acids and vitamin B1), whereas the capability to correctly rank the food group intakes was considered as good for 8 food groups, acceptable for 7 food groups and poor for 3 food groups (unsweetened beverages, fats, and sauces).

We found a median weighted κ of 0.32 (from 0.19 to 0.44) for nutrients (Table 4) and 0.27 (from –0.01 to 0.43) for food groups (Table 5). The level of agreement from weighted κ was considered as moderate for 8 nutrients, fair for 26 nutrients, and poor for 5 nutrients (MUFAs, *n*−3 fatty acids, *n*−6 fatty acids, vitamin E, and selenium). The level of agreement from weighted κ was considered as moderate for 2 food groups, fair for 12 food groups, and poor for 4 food groups (unsweetened beverages, milk and dairy products, fats and sauces).

Sensitivity analyses excluding participants who reported extreme energy intakes produced similar results (Supplemental Tables 3 and 4).

## Discussion

The 181-item semiquantitative FFQ that we developed for use among the adult population in Reunion Island demonstrated a moderate to good level of validity for ranking food and nutrient intakes, and a good level of reproducibility. To the best of our knowledge, this is the first culture and context-specific FFQ that has been developed and validated specifically for Reunion Island.

We found that the second application of the CARI FFQ globally produced higher energy, nutrient, and food group estimates compared with those of the DRs. Such results were expected, as FFQs are known to reflect higher estimates than the reference method, which are usually repeated 24-h DRs [9,10], a tendency to overestimate that is all the more important when FFQs contain >100 items [30,31]. Like the FFQ, 24-h DRs are prone to measurement error. Nevertheless, the major type of measurement error in 24-h DRs is random, whereas the major type is systematic in FFQ, and in 24-h DRs rely on specific memory, whereas FFQ relies on generic memory [[7], [8], [9]]. In a meta-analysis considering the reproducibility of FFQs to examine nutrient intake among healthy populations, Cui et al. [32] found that the pooled crude intraclass correlation coefficients ranged from 0.50 to 0.80 for macronutrients and 0.50 to 0.72 for micronutrients, and that the pooled crude Spearman correlation coefficients ranged from 0.55 to 0.85 for macronutrients, and from 0.57 to 0.83 for micronutrients. These results are quite similar to what we obtained in our study, as well as in another study using a 1-mo FFQ and published after Cui’s meta-analysis, with crude correlations varying from 0.37 to 0.70 for nutrients in Kenyan adults [33]. In a systematic review of studies for the validation of semiquantitative FFQ that assesses food intake in adults, Sierra-Ruelas et al. [10] reported that energy-adjusted and deattenuated correlation coefficients for nutrients ranged from −0.03 to 1.00, and that crude correlation coefficients ranged from −0.01 to 1.00 for food groups. Although the range of these values is quite wide, this reflects the breadth of our results with energy-adjusted and deattenuated correlation coefficients ranging from 0.13 to 0.67 for nutrients and crude correlation coefficients ranging from –0.02 to 0.71 for food groups. These results are quite similar to what other studies using a 1-mo FFQ and published after Sierra-Ruelas’ review, with energy-adjusted and deattenuated correlations varying from −0.18 to 0.61 for nutrients in Kenyan adults [33], and from 0.06 to 0.62 for nutrients in adult Emiratis [34].

Because the CARI observation center aims to conduct a large-scale survey representative of the population of Reunion Island about diet and nutrition, several methodological choices were made regarding the development of the CARI FFQ and the design of the reproducibility and validity study. To minimize nonresponse of the future large-scale survey, which is often higher among younger individuals and those with lower educational attainment, we adopted a 1-mo reference period to reduce respondent burden, FFQs covering long reference periods placing greater cognitive demands on respondents than those with shorter periods [7,8]. To further encourage participation, the FFQ was administered by interviewers, as self-administered modes are associated with higher nonresponse in health surveys [35]. When developing the CARI FFQ, we closely considered food culture, dietary practices, and nutritional issues on Reunion Island. Although this involved adding local foods and recipes, we also reconsidered whether certain food items should be more or less detailed, especially regarding sugar content. We found acceptable to good validity levels for sweetened beverages, and good validity levels for added sugars. This result is particularly important in the context of Reunion Island, where there is a high prevalence of type 2 diabetes [4,36], and considering that sweetened beverages and added sugars contribute to chronic disease risk through weight gain and through development of risk factors precipitated by adverse glycemic effects [37]. However, low validity was evident for some nutrients (e.g., PUFAs) and food groups (e.g., unsweetened beverages and fats). Improving the CARI FFQ could involve expanding the range of frequency options for unsweetened beverages, as done in other FFQs [38], particularly for items from this group, such as mineral and tap water, which may be consumed multiple times throughout the day [39]. Another improvement could involve adding more details to some food items according to their lipid profiles, but this could also lead to an overestimation of intake [30,31] and increase the duration of filling out the questionnaire, leading to an increase in the burden for the respondent [9]. As a result, precautions must be taken when analyzing these nutrients and food groups.

One of the strengths of this study is its design to test validity and reproducibility based on the guidance provided by the literature [7,9]. Another strength is the balanced and diversified sample in terms of age, gender, socioeconomic and location, which attempts to reflect as well as possible the composition of the Reunion Island population. The main limitation of our study might rely on the choice of our reference method to validate the CARI FFQ. As assessing the true validity of an FFQ would require measuring with great accuracy the habitual diet chosen by free-living people, the researchers could only assess relative validity by comparing the FFQ to another diet assessment method with its own limitations [28]. Although nutritional biomarkers provide intake estimates independent of self-reported data and are therefore less susceptible to under-reporting or recall bias, their cost was incompatible with the resources of our study, and their invasive nature remains a major limitation [9]. In the case of this study, as in many other relative validation studies [10], we used the repeated 24-h DRs as the reference method. Intrusion (i.e., food or beverage reported but not consumed), omission (i.e., food or beverage consumed but not reported), misclassification, and portion misestimation are known to contribute to dietary intake measurement error in short-term dietary assessment instruments like 24-h DRs [40]. We make the assumption that the repeated 24-h DRs designed and validated for adults living in mainland France [20,41] are not best suited to Reunion Island’s context in terms of portion sizes and prompts for accompanying food items [41], leading to a possible underestimation of dietary intakes like in the Guadeloupean and Martinican context [42]. As the accuracy of estimates obtained with 24-h recalls is thus questionable in these contexts, we chose to focus our validity study on the ability of the CARI FFQ for ranking food and nutrient intakes within a population. Another limitation of our study is that we were unable to rely on previous detailed dietary surveys to optimally define the items to be included in the CARI FFQ. Indeed, when such surveys exist, it is possible to select foods on the basis of their contribution to energy and nutritional intakes, generally by looking for a cumulative contribution of ≥80% of intakes [43,44]. Nevertheless, we were able to mobilize a wide range of data and knowledge (local dietitians, household budget survey, data on imports, agricultural production, and school canteen menus) to guide our choices in the selection of relevant foods. A last limitation of our study is the existence of a learning effect throughout the study that could improve quality in completing the second application of the CARI FFQ, especially with a short reference period of 1 mo. However, it is essential that the assessment period be the same for both FFQ and reference methods [9].

In conclusion, our results indicate that the CARI FFQ has a moderate to good level of validity for ranking nutrient and food intakes, and a good level of reproducibility, for use in the adult population in Reunion Island. Although it remains difficult to gauge its level of accuracy in measuring absolute intakes, the CARI FFQ is a valuable tool for ranking individuals according to their nutrient and food intakes. As a result, this FFQ could play an important role in identifying groups at risk of diet-related diseases. Similarly, by matching with appropriate databases, it will be possible to identify groups of participants whose diets present different levels of sustainability according to the nutritional, economic, and environmental dimensions. In addition, the good level of reproducibility means that the CARI FFQ is suitable for producing data for monitoring dietary behavior.

## Data availability

Data described in the manuscript, code book, and analytic code will be made available on request pending application and approval by the authors of the current study.

## Author contributions

The authors’ responsibilities were as follows – EOV, SE-D,CM: designed the study; EOV, SE-D, MT, CM: developed the food frequency questionnaire; BA: provided nutritional composition data and supported the used of the web-based diet record tool; SA, NN: trained dietitians and monitored the quality of the collection; SEB: analyzed data, drafted the figures and tables; EOV: drafted the manuscript; SE-D, SA, NN, MT, JG-B, LDC, MO, BA, CM: provided critical review; and all authors: read and approved the final manuscript.

## Funding

This study was part of the CARI project (observatoire des Comportements Alimentaires à la RéunIon; https://pepr-sams.fr/en/2024/03/26/cari-en/), supported by the French state, managed by the French National Research Agency (Agence Nationale de la Recherche, ANR) in the context of France 2030 (ANR-22-PESA-0002).

## Conflict of interest

The authors report no conflicts of interest.
